# Ketamine exerts a protective role in a cell-based model of major depressive disorder via the inhibition of apoptosis and inflammation and activation of the Krebs cycle

**DOI:** 10.17305/bjbms.2019.4222

**Published:** 2020-02

**Authors:** Wenfei Zhang, Qian Sun, Lingling Jia, Ming Li

**Affiliations:** 1 Psychological Outpatient Department, Dezhou People’s Hospital, Dezhou, China; 2 Department of Pharmacy, Dezhou People’s Hospital, Dezhou, China; 3 Department of Orthopedics, Dezhou People’s Hospital, Dezhou, China

**Keywords:** Ketamine, major depressive disorder, MDD, apoptosis, Krebs cycle, corticosterone, nuclear factor-κB, NF-κB, inflammation, cytokines

## Abstract

Major depressive disorder (MDD) is one of the most common psychiatric disorders characterized by major depressive episodes. Although great efforts have been made to develop antidepressant drugs that target the monoaminergic system, these drugs are effective in only approximately 50% of MDD patients. In this study, we established a model of depression in PC12 cells using corticosterone to investigate the effect of ketamine and nuclear factor-κB (NF-κB) on the cell viability, apoptosis, levels of pro-inflammatory cytokines, apoptosis-related molecules, and enzymes of the Krebs cycle. PC12 cells were divided into control (no treatment, NC), ketamine treatment (KT), ketamine treatment with the inhibition of NF-κB (KI), and ketamine treatment with the overexpression of NF-κB (KO) group. Blood serum samples were collected from patients with MDD (n = 10) and healthy controls (n = 10) between 2015 and 2017. Ketamine significantly increased the viability and decreased the apoptosis of PC12 cells in KT and KI vs. NC group, but not in KO group. The levels of anti-apoptotic molecules and Krebs cycle enzymes were significantly increased in KI vs. KT group, while the levels of pro-apoptotic molecules and pro-inflammatory cytokines were decreased in KI vs. KT group. In addition, the levels of pro-inflammatory cytokines in the serum of MDD patients were significantly increased. The antidepressant effect of ketamine was enhanced in KI and reduced in KO group. Our results indicate that ketamine exerts its antidepressant effect via the inhibition of apoptosis and inflammation and the activation of the Krebs cycle in PC12 cells. NF-κB might be a potential therapeutic target in MDD.

## INTRODUCTION

Major depressive disorder (MDD) is one of the most common psychiatric disorders characterized by major depressive episodes. Different factors are associated with MDD, such as physical changes in the brain, changes in neurotransmitters and hormones, and genetic changes. According to a WHO report, depression is expected to be the most common cause of nonfatal diseases by 2030 [1]. Moreover, 20–25% of depressed patients have a history of suicide attempts [2]. Since MDD was first recognized two decades ago, great efforts have been made to develop antidepressant drugs that target the monoaminergic system. However, these drugs are effective in only approximately 50% of MDD patients, and relief may take several weeks. In addition, some patients with MDD do not comply with current antidepressant treatments [3] and other experience side effects from the antidepressants [4]. The financial burden of depression treatment remains high each year both for public health systems and patients. Thus, an effective treatment for depression is imperative.

Pro-inflammatory cytokines, including interleukin (IL)-1β, IL-2, IL-6, and tumor necrosis factor (TNF)-α, play an important role in the response of antidepressant-resistant patients to the drugs; the concentrations of these cytokines were significantly higher in these patients compared with patients without depression [5,6]. The concentration of pro-inflammatory cytokines in the plasma of patients with major depression is significantly associated with the concentration of glutamate in the basal ganglia, and both processes are implicated in the development of depressive behavior [7]. High levels of corticosterone (CORT) induce depression-like behavior in mice, and the treatment of PC12 cells with high concentrations of CORT induces pathological changes that mimic those observed in depressive disorders [8,9].

The two main apoptotic pathways are the extrinsic or death receptor pathway and the intrinsic or mitochondrial pathway. The B-cell lymphoma 2 (Bcl-2) family consists of a number of proteins, which have critical roles in the regulation of apoptosis. Bcl-2 is an anti-apoptotic member of the Bcl-2 family, which inhibits the intrinsic apoptotic pathway in cells and thus promotes oncogenesis [10]. Other proteins in the Bcl-2 family, such as Bcl-2-associated X (Bax), Bcl-2-associated death promoter (Bad), and Bcl-2 homologous antagonist/killer (Bak) perform pro-apoptotic functions. Bax is located on the outer membrane of mitochondria and exerts its effects by inducing the release of cytochrome c [11]. Bak contains a C-terminal transmembrane domain that allows Bak to anchor to the outer mitochondrial membrane [12]. Bcl-2 homology (BH)3-only proteins directly interact with Bax/Bak to trigger conformational changes and initiate apoptosis [13].

Ketamine is an antagonist of glutamate receptors, and more attention has been paid to the antidepressant function of ketamine [14]. An increasing number of studies have found that ketamine is effective for patients with treatment-resistant major depression, and rapid patient response to the first ketamine infusion is predictive of a sustained ketamine effect after subsequent infusions [15,16]. In one study, patients with treatment-resistant MDD showed an improved response to positive emotion after standard ketamine treatment [17]. Another study investigated the effects of ketamine on working memory performance in relation to emotional content and emotional valence of stimuli in healthy subjects. They demonstrated reduced activation in the left and right insula and in the right dorsolateral prefrontal cortex (DLPFC) after ketamine treatment, especially under a negative emotion [18].

The transcription factor NF-κB (nuclear factor kappa-light-chain-enhancer of activated B cells) plays an important role in neuronal survival and synaptic plasticity, in addition to its role in the regulation of inflammation and immune responses [19]. NF-κB regulates the expression of multiple immune genes, especially those encoding pro-inflammatory cytokines and chemokines, which are important for the development of the immune system. Thus, we speculated that a change in the expression of NF-κB might be related to the therapeutic effects of ketamine in the treatment of MDD and that NF-κB may represent a new therapeutic target for this disease.

In this study, we established a model of depression in PC12 cells using CORT to investigate the effect of ketamine and NF-κB on the cell viability, apoptosis, levels of pro-inflammatory cytokines, apoptosis-related molecules, and enzymes of the Krebs cycle. Ketamine treatment increased the viability and decreased apoptosis of PC12 cells. The levels of anti-apoptotic molecules and Krebs cycle enzymes were increased after ketamine treatment, while the levels of pro-apoptotic molecules and pro-inflammatory cytokines were decreased. In addition, the levels of pro-inflammatory cytokines in the serum of MDD patients were increased. Our results indicate that ketamine exerts its antidepressant effect via the inhibition of apoptosis and inflammation and the activation of the Krebs cycle in PC12 cells. Interestingly, the antidepressant effect of ketamine was enhanced in PC12 cells treated with ketamine combined with the inhibition of NF-κB and reduced in cells that underwent ketamine treatment combined with the overexpression of NF-κB.

## MATERIALS AND METHODS

### Reagents

RPMI 1640 medium (61870-036) and fetal bovine serum [FBS] (10099141) were purchased from Gibco (USA, NY). CORT and neferine (HY-N0441) were purchased from MedChem Express (NJ, USA). The GenElute Gel Extraction Kit (NA1111) was purchased from Sigma-Aldrich (Darmstadt, Germany). BamHI (R0136S) and XhoI (R0146S) were purchased from NEB (NY, USA). Lipofectamine 3000 Transfection Reagent (L3000015) was purchased from Invitrogen (CA, USA). G418 (G8161) was purchased from Solarbio (Beijing, China). RNApure Tissue and Cell Kit (CW0584), HiFiScript cDNA Synthesis Kit (CW2569), and Super TaqMan Mixture (CW2698) were obtained from Cwbiotech (Beijing, China). Antibodies against Bcl-2 (ab32124), Bax (ab32503), Bad (ab32445), p53 (ab26), Bak (ab32371), succinate-CoA ligase GDP-forming beta subunit [SUCLG2] (ab96172), aconitase 2 [ACO2] (ab110321), malate dehydrogenase 1 [MDH1] (ab180152), citrate synthase [CS] (ab96600), isocitrate dehydrogenase [IDH] (ab172964), NF-κB p65 (ab16502), and glyceraldehyde 3-phosphate dehydrogenase [GAPDH] (ab8245) were purchased from Abcam (Massachusetts, US). ELISA kits for IL-1β (ab100562), IL-2 (ab174444), IL-6 (ab46027), TNF-α (ab181421), interferon-γ [IFN-γ] (ab46025), and granulocyte colony-stimulating factor [G-CSF] (ab100524) were purchased from Abcam. Rabbit and mouse secondary antibodies were purchased from ECL.

### Cell culture

PC12 cell line (TCR 9) was purchased from the cell bank of the Typical Culture Preservation Committee of the Chinese Academy of Science. The cells were seeded into a 100-mm plate containing RPMI 1640 medium with 10% FBS and cultured in a humid atmosphere with 5% CO_2_ at 37°C until they reached a confluency of 70–80%. Then, the cells were centrifuged at 94 g for 10 minutes at room temperature. The cell pellet was collected and resuspended in 8 mL of medium, seeded into four new 100-mm plates, and cultured for 24 hours until they reached 60–70% confluency. A model of depression was established in PC12 cells by applying 500 µM CORT for 36 hours, according to a previous study [20]. An NF-κB inhibition cell model was constructed using neferine. NF-κB cDNA was synthesized using the following primers: forward, 5′-GAAGAAGCGAGACCTGGAG-3′; reverse, 5′-TCCGGAACACAATGGCCAC-3′. The cDNA of NF-κB and pcDNA3.1-3×Flag vector were digested with BamHI and XhoI and ligated overnight. After 48 hours, the pcDNA3.1-3×Flag-NF-κB overexpression vector was transfected into PC12 cells using Lipofectamine 3000 Transfection Reagent. The screening of vector(+) cells was performed using 1000 µg/mL G418. After the cell models were established, the cells were divided into four groups, as follows: control (no treatment, NC), ketamine treatment (KT), ketamine treatment with the inhibition of NF-κB (KI), and ketamine treatment with the overexpression of NF-κB (KO). The cells in experimental groups were treated with 0.2 µg/mL ketamine for 3 hours [21].

### MTT assay

The MTT assay was performed according to a previous study [22]. Briefly, the cells were seeded into 96-well plates at a concentration of 1×10^4^ cells per well. The cells were treated and grouped as described above. Then, the cells were washed with sterile phosphate-buffered saline (PBS). After centrifugation, MTT assay was performed in triplicate. The cells were incubated with 5 mg/ml MTT for 3 hours. The optical density (OD) at 490 nm was measured with a SpectraMax Series Microplate Reader (Sunnyvale, CA, USA). The viability rate was calculated as: (OD_Treatment_-OD_Blank_)/(OD_Control_-OD_Blank_).

### Ethical statement and tissue samples

This study was approved by the Medical Ethics and Human Clinical Trial Committee of Dezhou People’s Hospital. The experiment was carried out in compliance with the Helsinki Declaration. Each patient in this study gave written informed consent prior to recruitment. The patients were diagnosed with MDD using Hamilton Depression Scale (HAM-D score ≥23) [23,24]. The exclusion criteria were are follows: 1) bipolar disorder and post-schizophrenic depression; 2) organic mental disorder with mental retardation caused by a life event; 3) pregnancy or breastfeeding in women; and 4) serious functional defects of the heart, liver, kidneys, or other major organs. The blood serum samples of healthy controls (n = 10) and patients with MDD (n = 10) were collected at Dezhou People’s Hospital between 2015 and 2017. Baseline characteristics of participants are listed in Table 1. None of the participants were smokers nor had a history of alcohol abuse. They also did not use antidepressant drugs at the time blood samples were collected. All methods were carried out in accordance with the approved guidelines. The blood samples of patients and healthy controls were collected in the morning after fasting for 8 hours and using sterile blood collection tubes.

**TABLE 1:**
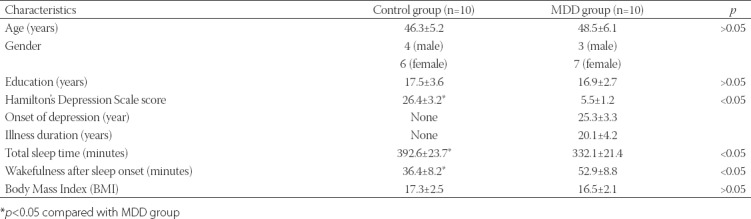
Baseline characteristics of patients with major depressive disorder (MDD) and healthy controls

### RNA extraction

RNA extraction from PC12 cells was performed according to the following protocol. Briefly, the cells were lysed with lysis buffer and centrifuged at 13,523 g for 5 minutes. Next, the supernatant was collected, loaded into spin columns, and centrifuged at 13,523 g for 1 minute. The total RNA was washed with wash buffer, eluted in elution buffer, and stored at -80°C until used.

### Reverse transcription quantitative PCR (RT-qPCR)

RT-qPCR was performed using the following protocol. Briefly, the reaction mixture was prepared according to the manufacturer’s instructions and incubated at 42°C for 15 minutes and then at 85°C for 5 minutes. Then, cDNA was prepared and mixed with the reaction mixture. The PCR reaction was incubated for 10 minutes at 95°C, followed by 40 cycles at 95°C for 15 seconds and at 60°C for 1 minute. The following primers were used: TNF-α forward: 5′-ATGAGCACGGAAAGCATGATC-3′, reverse: 5′-AGTAGACCTGCCCGGACTCCG-3′; IL-1β forward: 5′-AAATGCCTCGTGCTGTCTGACC-3′, reverse: 5′-GGTGGGTGTGCCGTCTTTCATC-3′; IL-2 forward: 5′-AAGGAAACACAGCAGCACCT-3′, reverse: 5′-CACAGTTGCTGGCTCATCAT-3′; IL-6 forward: 5′-AGCCCACCAGGAACGAAAG-3′, reverse: 5′-GGAAGGCAGTGGCTGTCAA-3′; IFN-γ forward: 5′-GTGCTTAGCCTGGTATTCATCTG-3′, reverse: 5′-ACTTTTCCTGGATTGTCTTCGG-3′; G-CSF forward: 5′-ATTAAGCCTGGAGGGCCTTG-3′, reverse: 5′-GAGTGACTCCCCTCTCTGGT-3′. The information on each gene was acquired from PubMed and the primers were designed using BLAST (https://blast.ncbi.nlm.nih.gov/Blast.cgi). The threshold cycle (Ct) was calculated by normalizing the fluorescence signal with the blank control. The quantity of target gene in the starting sample was determined in relation to the Ct values of serial dilutions of positive control. Glyceraldehyde 3-phosphate dehydrogenase (GAPDH) was used as an internal reference, and the quantification of target genes was normalized to relative GAPDH levels.

### Western blotting

NF-κB p65 and proteins related to apoptosis and the Krebs cycle were analyzed in PC12 cells by Western blotting, according to a previous study [25]. Briefly, the cells were lysed with lysis buffer and centrifuged at 13,523 g for 10 minutes. Then, the supernatant was collected and denatured with loading buffer. A bicinchoninic acid (BCA) assay was used to determine the concentration of proteins in each group. The same amount of each protein sample (60 µg) was separated using 10% sodium dodecyl sulfate–polyacrylamide gel electrophoresis (SDS-PAGE) electrophoresis and was transferred onto a 0.22 µm nitrocellulose membrane using a semi-dry electro-blotter. The membranes were incubated with primary antibodies (1:1000) overnight at 4°C, followed by an incubation with secondary antibody (1:5000) for 1 hour at room temperature. The protein concentration was detected using the ECL immunoblotting reagent. The gray values of the bands were quantified with Scion Image software and normalized to GAPDH.

### Flow cytometry

The cells in each group were first seeded into a 100 mm plate at a concentration of 1×10^6^. After being treated as previously described, the cells were digested with trypsin and washed with pre-cooled PBS. Next, the cells were incubated with propidium iodide (PI) and Annexin-V for 15 minutes, and the apoptotic cells were counted by FACScan flow cytometry (Becton Dickinson, Heidelberg, Germany).

### Enzyme-linked immunosorbent assay (ELISA)

Pro-inflammatory cytokines in serum samples of MDD patients and cultured medium of PC12 cells were detected using ELISA, according to the protocol. Briefly, the serum collected from the patients was centrifuged at 1,150 g for 10 minutes (4°C), and the serum samples and cultured medium of cells were then added to each well of a 96-well plate. After the addition of reaction buffer and incubation at room temperature for 5 minutes, the integral OD (IOD) at 450 nm was detected using a microplate reader. Blinded laboratory personnel performed the ELISA assays, and each sample was replicated three times.

### Statistical analysis

The data are presented as means ± SD, and each experiment was repeated in triplicate. The differences between the groups were analyzed using one-way ANOVA followed by Tukey’s post-hoc test. A value of *p* < 0.05 was considered statistically significant.

## RESULTS

### Detection of PC12 cell viability by MTT assay

**FIGURE 1:**
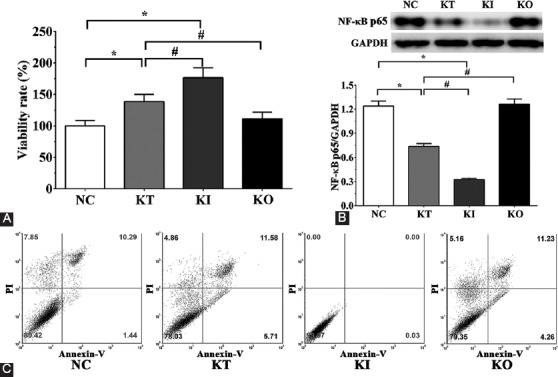
(A) Cell viability by MTT assay; (B) NF-κB p65 expression by Western blotting; (C) apoptosis by flow cytometry in PC12 treated with corticosterone. Ketamine significantly increased the viability and decreased the apoptosis of PC12 cells in KT and KI vs. NC group, but not in KO group. The experiments were repeated three times. The results are expressed as mean ± SD. *p < 0.05 vs. NC group. #p < 0.05 vs. KT group. NC: No treatment control group; KT: Ketamine treatment; KI: Ketamine treatment with the inhibition of NF-κB; KO: Ketamine treatment with the overexpression of NF-κB. NF-κB: Nuclear factor kappa-light-chain-enhancer of activated B cells.

As shown in Figure 1, the viability rates were 100.00 ± 8.23, 138.46 ± 11.24, 176.43 ± 15.77, and 110.97 ± 10.68 in NC, KT, KI, and KO group, respectively. Compared with NC group, the viability rate in KT and KI groups was significantly increased (*p* < 0.05). Compared with KT group, the viability rate was significantly increased in KI group (*p* < 0.05) and was significantly decreased in KO group (*p* < 0.05).

### Detection of NF-κB p65 in PC12 cells by Western blotting

The expression levels of NF-κB p65 were 1.24 ± 0.06, 0.74 ± 0.04, 0.33 ± 0.02, and 1.26 ± 0.06, respectively in NC, KI, KT, and KO groups (Figure 1). The expression of NF-κB p65 was significantly decreased in KT and KI groups compared with NC group (*p* < 0.05). NF-κB p65 expression was significantly decreased in KI group and significantly increased in KO group compared with KT group (*p* < 0.05).

### Detection of apoptosis in PC12 cells by flow cytometry

The number of apoptotic cells was 18.32 ± 0.82, 14.34 ± 0.65, 0.05 ± 0.01, and 21.63 ± 1.23 in NC, KI, KT, and KO groups, respectively (Figure 1). Compared with NC group, the number of apoptotic cells was significantly decreased in KT and KI groups (*p* < 0.05) and was significantly increased in KO group (*p* < 0.05). Compared with KT group, the number of apoptotic cells was significantly decreased in KI group (*p* < 0.05) but was significantly increased in KO group (*p* < 0.05).

### Detection of apoptosis-related proteins in PC12 cells by Western blotting

**FIGURE 2:**
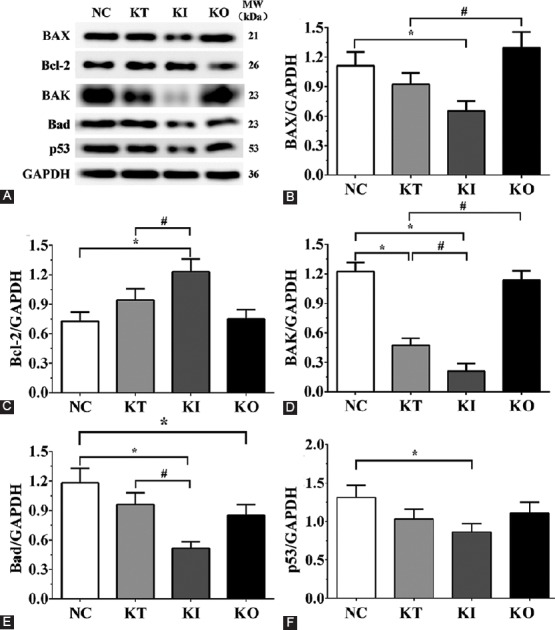
Expression of apoptosis-related molecules by Western blotting in PC12 cells treated with corticosterone. (A) Expression levels of Bax, Bcl-2, Bak, Bad, and p53; (B-F) quantitative analysis of Western blotting results. The experiments were repeated three times. The levels of anti-apoptotic molecules were significantly increased in KI vs. KT group, while the levels of pro-apoptotic molecules were decreased. The results are expressed as mean ± SD. *p < 0.05 vs. NC group. #p < 0.05 vs. KT group. GAPDH was used as the internal control. NC: No treatment control group; KT: Ketamine treatment; KI: Ketamine treatment with the inhibition of NF-κB; KO: Ketamine treatment with the overexpression of NF-κB. Bax: Bcl-2-associated X; Bcl-2: B-cell lymphoma 2; Bak: Bcl-2 homologous antagonist/killer; Bad: Bcl-2-associated death promoter; GAPDH: Glyceraldehyde 3-phosphate dehydrogenase; NF-κB: Nuclear factor kappa-light-chain-enhancer of activated B cells.

As shown in Figure 2, the expression levels of Bax in NC, KT, KI, and KO groups were 1.21 ± 0.12, 0.46 ± 0.08, 0.18 ± 0.03, and 1.12 ± 0.10, respectively. Compared with NC group, the expression of Bax was significantly decreased in KI group (*p* < 0.05). Compared with KT group, the expression of Bax was significantly increased in KO group (*p* < 0.05) and significantly decreased in KI group (*p* < 0.05). The expression levels of Bad in NC, KT, KI, and KO groups were 1.18 ± 0.15, 0.96 ± 0.12, 0.52 ± 0.06, and 0.85 ± 0.11, respectively. Compared with NC group, the expression of Bad was significantly decreased in KT and KI groups (*p* < 0.05). The expression of Bad was significantly decreased in KI compared with KT group (*p* < 0.05). Bcl-2 expression levels were 0.73 ± 0.09, 0.94 ± 0.12, 1.03 ± 0.13, and 0.75 ± 0.09, respectively in NC, KI, KT, and KO groups. Compared with NC group, the expression of Bcl-2 was significantly increased in KI group (*p* < 0.05). Compared with KT group, the expression of Bcl-2 was significantly increased in KI group (*p* < 0.05). The expression levels of p53 in NC, KI, KT, and KO groups were 1.31 ± 0.16, 1.03 ± 0.13, 0.86 ± 0.11, and 1.11 ± 0.14, respectively. Compared with NC group, the expression of p53 was significantly increased in KI group (*p* < 0.05). The expression levels of Bak in NC, KI, KT, and KO groups were 1.22 ± 0.08, 0.54 ± 0.06, 0.21 ± 0.03, and 1.16 ± 0.10, respectively. Compared with NC group, the expression of Bak was significantly decreased in KI and KT groups (*p* < 0.05). The expression of Bak was significantly increased in KO group and significantly decreased in KI group compared with KT group (*p* < 0.05).

### Detection of enzymes of the Krebs cycle by Western blotting

**FIGURE 3:**
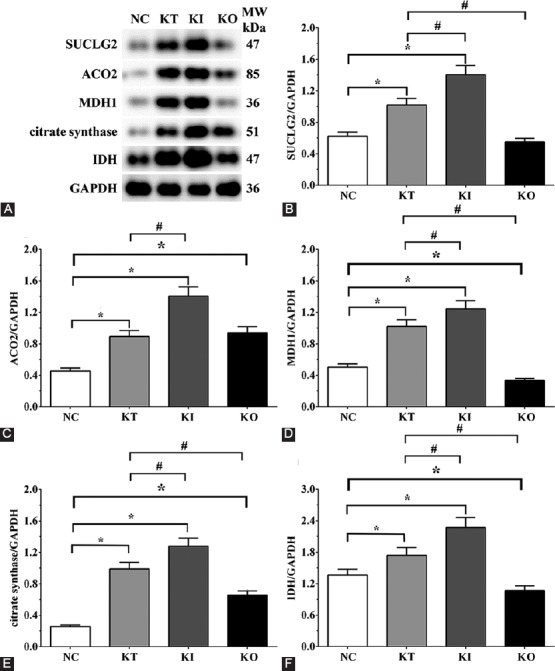
Expression of key enzymes of the Krebs cycle by Western blotting in PC12 treated with corticosterone. (A) Expression levels of SUCLG2, ACO2, MDH1, CS, and IDH; (B-F) quantitative analysis of Western blotting results. The levels of Krebs cycle enzymes were significantly increased in KI vs. KT group. The experiments were repeated three times. The results are expressed as mean ± SD. *p < 0.05 vs. NC group. #p < 0.05 vs. KT group. GAPDH was used as the internal control. NC: No treatment control group; KT: Ketamine treatment; KI: Ketamine treatment with the inhibition of NF-κB; KO: Ketamine treatment with the overexpression of NF-κB. SUCLG2: Succinate-CoA ligase GDP-forming beta subunit; ACO2: Aconitase 2; MDH1: Malate dehydrogenase 1; CS: Citrate synthase; IDH: Isocitrate dehydrogenase; NF-κB: Nuclear factor kappa-light-chain-enhancer of activated B cells; GAPDH: Glyceraldehyde 3-phosphate dehydrogenase.

As shown in Figure 3, the expression levels of SUCLG2 were 0.62 ± 0.05, 1.02 ± 0.08, 1.40 ± 0.12, and 0.55 ± 0.05, respectively in NC, KI, KT, and KO groups. Compared with NC group, the expression of SUCLG2 in KT and KI groups was significantly increased (*p* < 0.05). The expression of SUCLG2 was significantly increased in KI group (*p* < 0.05) and significantly decreased in KO group (*p* < 0.05), compared with KT group. The expression levels of ACO2 were 0.45 ± 0.04, 0.89 ± 0.07, 1.41 ± 0.12, and 0.94 ± 0.08, respectively in NC, KI, KT, and KO groups. The expression of ACO2 was also significantly increased in KT, KI, and KO groups (*p* < 0.05) compared with NC group. Compared with KT group, the expression of ACO2 was significantly increased in KI group (*p* < 0.05). The expression levels of MDH1 were 0.50 ± 0.04, 1.02 ± 0.09, 1.24 ± 0.10, and 0.33 ± 0.03, respectively in NC, KI, KT, and KO groups. Compared with NC group, the expression of MDH1 was significantly increased in KT and KI groups (*p* < 0.05) and significantly decreased in KO group (*p* < 0.05). The expression of MDH1 was significantly increased in KI group (*p* < 0.05) and significantly decreased in KO group (*p* < 0.05), compared with KT group. The expression levels of CS were 0.26 ± 0.02, 0.99 ± 0.08, 1.28 ± 0.11, and 0.65 ± 0.05, respectively in NC, KI, KT, and KO groups. The expression of CS was significantly increased in the experimental groups compared with NC group (*p* < 0.05). Moreover, CS expression was significantly increased in KI group (*p* < 0.05) and decreased in KO group (*p* < 0.05), compared with KT group. The expression levels of IDH were 1.36 ± 0.11, 1.74 ± 0.15, 2.27 ± 0.19, and 1.07 ± 0.09, respectively in NC, KI, KT, and KO groups. The expression of IDH was significantly increased in KT and KI groups (*p* < 0.05) and significantly decreased in KO group (*p* < 0.05), compared with NC group. IDH expression was significantly increased in KI group (*p* < 0.05) and significantly decreased in KO group (*p* < 0.05) compared with KT group.

### Detection of pro-inflammatory cytokines in PC12 cells by RT-qPCR

**FIGURE 4:**
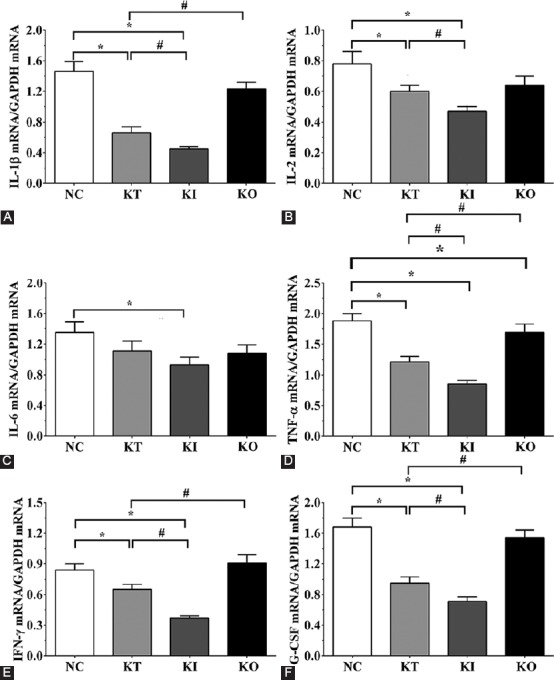
Expression of pro-inflammatory cytokines by RT-qPCR in PC12 cells treated with corticosterone. Expression levels of (A) IL-1β; (B) IL-2; (C) IL-6; (D) TNF-α; (E) IFN-γ; and (F) G-CSF. The levels of pro-inflammatory cytokines were significantly decreased in KI vs. KT group. The experiments were repeated three times. The results are expressed as mean ± SD. *p < 0.05 vs. NC group. #p < 0.05 vs. KT group. GAPDH was used as the internal control. NC: No treatment control group; KT: Ketamine treatment; KI: Ketamine treatment with the inhibition of NF-κB; KO: Ketamine treatment with the overexpression of NF-κB. IL: Interleukin; TNF-α: Tumor necrosis factor alpha; IFN-γ: Interferon gamma; G-CSF: Granulocyte colony-stimulating factor; GAPDH: Glyceraldehyde 3-phosphate dehydrogenase; NF-κB: Nuclear factor kappa-light-chain-enhancer of activated B cells; RT-qPCR: Reverse transcription quantitative PCR.

As shown in Figure 4, the expression levels of TNF-α in NC, KT, KI, and KO groups were 1.88 ± 0.12, 1.21 ± 0.09, 0.85 ± 0.06, and 1.69 ± 0.14, respectively. Compared with NC group, the expression of TNF-α was significantly decreased in KT and KI groups (*p* < 0.05). Compared with KT group, TNF-α expression was significantly decreased in KI group (*p* < 0.05) and was significantly increased in KO group (*p* < 0.05). The expression levels of IL-1β were 1.46 ± 0.13, 0.66 ± 0.08, 0.45 ± 0.03, and 1.23 ± 0.09 in NC, KI, KT, and KO groups, respectively. Compared with NC group, the expression of IL-1β was significantly decreased in KT and KI groups (*p* < 0.05). Compared with KT group, the expression of IL-1β was significantly decreased in KI group (*p* < 0.05) and was significantly increased in KO group (*p* < 0.05). The expression levels of IL-2 in NC, KI, KT, and KO groups were 0.98 ± 0.08, 0.65 ± 0.04, 0.47 ± 0.03, and 0.84 ± 0.06, respectively. Compared with NC group, the expression of IL-2 was significantly decreased in KT and KI groups (*p* < 0.05). IL-2 expression was significantly decreased in KI group compared with KT group (*p* < 0.05). The expression levels of IL-6 were 1.35 ± 0.14, 1.11 ± 0.13, 0.93 ± 0.10, and 1.08 ± 0.11, respectively in NC, KI, KT, and KO groups. The expression of IL-6 was significantly decreased in KI group compared with NC group (*p* < 0.05) but was not significantly different compared with KT group. IL-6 expression was not significantly different in KO group compared with NC or KT groups. The expression levels of IFN-γ in NC, KI, KT, and KO groups were 0.84 ± 0.06, 0.65 ± 0.05, 0.37 ± 0.02, and 0.91 ± 0.08, respectively. The expression levels of G-CSF were 1.68 ± 0.12, 0.95 ± 0.08, 0.71 ± 0.06, and 1.54 ± 0.10, respectively in NC, KI, KT, and KO groups. Compared with NC group, the expression levels of IFN-γ and G-CSF in KT and KI groups were significantly decreased (*p* < 0.05) but were not significantly different in KO group. IFN-γ and G-CSF levels were significantly decreased in KI group (*p* < 0.05) and significantly increased in KO group (*p* < 0.05) compared with KT group.

### Detection of pro-inflammatory cytokines in the serum of patients with MDD and in cultured medium of PC12 cells by ELISA

**FIGURE 5:**
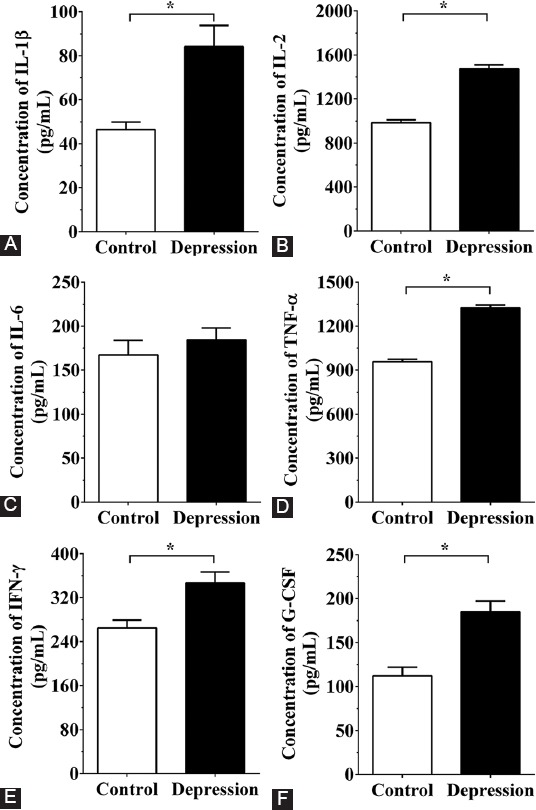
Concentration of pro-inflammatory cytokines by ELISA in the serum samples of patients with MDD (n = 10) and healthy controls (n = 10). Concentrations of (A) IL-1β; (B) IL-2; (C) IL-6; (D) TNF-α; (E) IFN-γ; and (F) G-CSF. The concentration of pro-inflammatory cytokines in the serum of MDD patients was significantly increased. The experiments were repeated three times. The results are expressed as mean ± SD. *p < 0.05 vs. control group. MDD: Major depressive disorder; IL: Interleukin; TNF-α: Tumor necrosis factor alpha; IFN-γ: Interferon gamma; G-CSF: Granulocyte colony-stimulating factor; GAPDH: Glyceraldehyde 3-phosphate dehydrogenase; NF-κB: Nuclear factor kappa-light-chain-enhancer of activated B cells; ELISA: Enzyme-linked immunosorbent assay.

The serum concentration of several cytokines was significantly increased in MDD vs. control group (Figure 5), as follows: IL-1β (84.11 ± 9.67 pg/mL vs. 46.41 ± 3.54 pg/mL), IL-2 (1479.13 ± 33.28 pg/mL vs. 985.47 ± 24.16 pg/mL), TNF-α (1323.47 ± 21.44 pg/mL vs. 956.43 ± 18.56 pg/mL), IFN-γ (346.92 ± 19.35 pg/mL vs. 264.78 ± 14.53 pg/mL), G-CSF (184.63 ± 12.45 pg/mL vs. 112.34 ± 9.51 pg/mL). On the other hand, IL-6 concentration was not significantly different between MDD patients (184.35 ± 13.48 pg/mL) and controls (167.12 ± 16.66 pg/mL).

**FIGURE 6:**
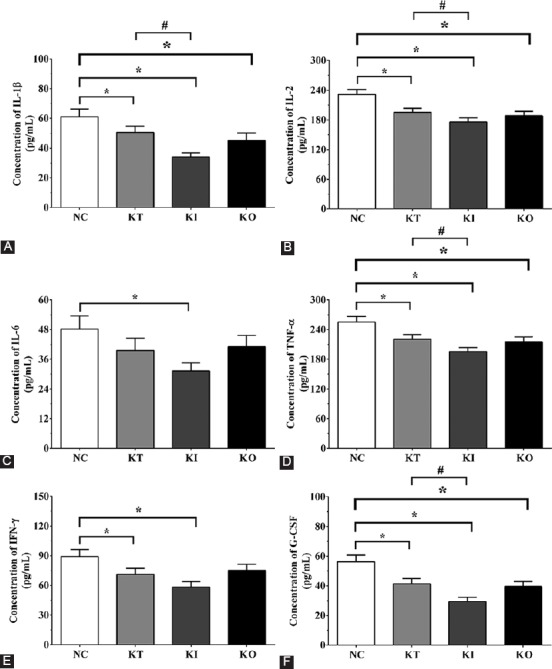
Concentration of pro-inflammatory cytokines by ELISA in the cultured medium of PC12 cells treated with corticosterone. Concentrations of (A) IL-1β; (B) IL-2; (C) IL-6; (D) TNF-α; (E) IFN-γ; and (F) G-CSF. The concentration of pro-inflammatory cytokines was decreased in KI vs. KT group. The experiments were repeated three times. The results are expressed as mean ± SD. *p < 0.05 vs. NC group. #p < 0.05 vs. KT group. NC: No treatment control group; KT: Ketamine treatment; KI: Ketamine treatment with the inhibition of NF-κB; KO: Ketamine treatment with the overexpression of NF-κB. IL: Interleukin; TNF-α: Tumor necrosis factor alpha; IFN-γ: Interferon gamma; G-CSF: Granulocyte colony-stimulating factor; GAPDH: Glyceraldehyde 3-phosphate dehydrogenase; NF-κB: Nuclear factor kappa-light-chain-enhancer of activated B cells; ELISA: Enzyme-linked immunosorbent assay.

The concentration of these cytokines in cultured medium of PC12 cells is shown in Figure 6. The concentration of IL-1β in the medium of PC12 cells was 61.2 ± 5.1, 50.5 ± 4.2, 34.1 ± 2.8, and 45.2 ± 5.0 pg/mL, respectively in NC, KT, KI, and KO groups. Compared with NC group, IL-1β concentration was significantly decreased in KT, KI, and KO groups (*p* < 0.05). It was also significantly decreased in KI compared with KT group (*p* < 0.05). The concentration of IL-2 was 231.3 ± 9.8, 195.2 ± 8.1, 175.6 ± 8.3, and 188.2 ± 8.9 pg/mL in NC, KI, KT, and KO groups. TNF-α concentration was 255.3 ± 11.2, 220.5 ± 9.6, 195.6 ± 8.3, and 215.3 ± 10.0 pg/mL in NC, KI, KT, and KO groups, respectively. The changes in the concentration of IL-2 and TNF-α were similar to that of IL-1β. The concentration of IL-6 was 48.2 ± 5.3, 39.6 ± 4.9, 31.3 ± 3.2, and 41.1 ± 5.5 pg/mL in NC, KI, KT, and KO groups. IL-6 concentration was significantly decreased in KI compared with NC group (*p* < 0.05). IFN-γ concentration was 89.2 ± 7.1, 71.1 ± 6.2, 58.3 ± 5.5, and 75.2 ± 6.1 pg/mL in NC, KI, KT, and KO groups. The concentration of IFN-γ was significantly decreased in KT and KI groups compared with NC group (*p* < 0.05). G-CSF concentration in the medium of PC12 cells was 56.2 ± 4.5, 41.3 ± 3.5, 29.5 ± 2.8, and 39.6 ± 3.3 pg/mL in NC, KI, KT, and KO groups. Compared with NC group, the concentration of G-CSF was significantly decreased in KT, KI, and KO groups (*p* < 0.05); it was significantly decreased in KI compared with KT group (*p* < 0.05).

## DISCUSSION

MDD is one of the most common psychiatric disorders characterized by major depressive episodes. Previous studies have found that ketamine is effective as an antidepressant, but the exact mechanism is not yet fully understood. In this study, we established a model of depression in PC12 cells using CORT and investigated the effect of ketamine and NF-κB on the cell viability, apoptosis, levels of pro-inflammatory cytokines, apoptosis-related molecules, and enzymes of the Krebs cycle. We found that ketamine increased the viability of PC12 cells, most probably via the inhibition of apoptosis and inflammation and the activation of the Krebs cycle. The inhibition of NF-κB enhanced and the overexpression of NF-κB reduced the antidepressant effect of ketamine in PC12 cells.

Apoptosis maintains the balance between cell proliferation and cell death and is also important for the recycling of cellular components [26]. We presumed that a reduced expression of anti-apoptotic Bcl-2 family members in the PC12 depression model would lead to the activation of caspase cascade, resulting in the apoptosis of PC12 cells. We further hypothesized that ketamine treatment combined with the inhibition of NF-κB expression could reverse that process. We found that the expression of pro-apoptotic factors Bax, Bak, and Bad was reduced in KT group and was even lower in KI group, in which NF-κB expression was inhibited. This shows that ketamine could reduce the apoptosis of CORT-treated PC12 cells and, therefore, may have a protective role in MDD. Extrinsic stress signals activate p53 which then regulates downstream molecules by transcriptional activation [27]. Activated p53 phosphorylates Bad in the cytosol [28], which is further cleaved by caspase-8 (a downstream molecule of the extrinsic apoptotic pathway); the cleaved Bad then translocates to mitochondria and activates apoptosis [29]. The expression of p53 was decreased in our PC12 cells treated with ketamine and was even more reduced in the cells treated with ketamine combined with the inhibition of NF-κB. The reduced levels of p53 in these cells indicate inhibited activation of the extrinsic apoptotic pathway after ketamine treatment. Overall, our results indicate that the inhibition of apoptosis was a critical step in the protective function of ketamine in the cell model of MDD.

The Krebs cycle consists of a series of chemical reactions that result in the release of stored energy in cells. The production of adenosine triphosphate (ATP) is critical for the survival of neurons. In the Krebs cycle, succinyl-CoA ligases (SUCLs) reversibly catalyze the conversion of succinyl-CoA and adenosine diphosphate (ADP) or guanosine diphosphate (GDP) into succinate and ATP or guanosine triphosphate (GTP), respectively. SUCLs are divided into three subtypes: ADP-forming SUCLA2, with high expression in the brain and skeletal muscles; GDP-forming SUCLG2, with high expression in the liver and kidneys; and GDP-forming SUCLG1, which is ubiquitously expressed in all tissues [30]. ACO2 catalyzes the interconversion of citrate into isocitrate via cis-aconitate as an intermediate, in a non-redox reaction. The activity of ACO2 is used as a biomarker of oxidative stress and ACO2 is suggested to act as an intramitochondrial sensor of redox status [31]. MDHs catalyze the conversion of oxaloacetate and malate utilizing the nicotinamide adenine dinucleotide (NAD)/NADH coenzyme system. MDH1 generates NADH by oxidizing malate into oxaloacetate and also reduces oxaloacetate into malate with the generation of NAD^+^ [32]. Citrate synthase (CS) catalyzes acetyl-CoA and oxaloacetate into citrate via the condensation of the acetyl group, which is the first step in the tricarboxylic acid (Krebs) and glyoxylate cycles. The activity of CS is inhibited by ATP, high redox levels, acetyl-CoA, succinyl-CoA, and citrate [33]. IDH is a key metabolic enzyme that catalyzes the oxidative decarboxylation of isocitrate into α-ketoglutarate with the reduction of NADP^+^ into NADPH [34]. These enzymes are critical in the Krebs cycle, which leads to the production of ATP. In this study, we found that ketamine increased the activity of SUCLG2, ACO2, MDH1, CS, and IDH, resulting in the production of ATP. Increased ATP induces cell repair mechanisms and enhances cell viability. In addition, the inhibition of NF-κB in our study enhanced the protective effect of ketamine in PC12 cells.

Depressive disorders is characterized by inflammation, as indicated by elevated concentrations of pro-inflammatory cytokines (e.g., IL-1β, IL-2, and IL-6), and it is characterized by cell-mediated immune (CMI) activation and Th1-like response, as suggested by increased production of IFN-γ. A previous study found that acute exposure to IFN-γ adenovector provokes long-lasting hedonic-like deficits [35]. However, the genetic deletion of IFN-γ or its receptor could induce depressive-like neurochemical, immunological, and behavioral effects [36]. In the current study, we found that the concentration of IFN-γ was increased in MDD patients; this indicates that the inflammatory process was activated in these patients, inducing the secretion of pro-inflammatory cytokines, such as IL-1β, IL-2, and IL-6. A previous study found that mitogen-stimulated lymphocytes increase IL-1β production in patients with depressive disorders and that a correlation exists between the production of IL-1β and the severity of depressive symptoms [37]. IL-6 regulates multiple pathological processes involved in depressive disorders, including the differentiation of specific T- and B-cells, acute phase response, activity of the hypothalamic-pituitary-adrenal (HPA) axis, and neuroprogression [38]. Patients with MDD who were administered T-cell cytokines including IL-2 exhibited remarkable alterations in depressive-like behavior, including depressed mood, fatigue, cognitive dysfunction, and sleep impairment, as well as in psychosis and delirium [39]. TNF-α plays an important role in the development of depressive disorders [40]. Patients with depressive disorders have high levels of inflammatory markers and mediators, including TNF-α. An animal study showed that long-term zeaxanthin treatment decreases the levels of IL-6, IL-1β, and TNF-α and ameliorates diabetes-associated anxiety and depression in diabetic rats [41]. G-CSF is a hematopoietic cytokine with neurotrophic and anti-apoptotic activities. G-CSF significantly decreases amyloid burden, promotes hippocampal neurogenesis, and improves spatial learning in a mouse model of Alzheimer’s disease [42]. In our study, the concentration of pro-inflammatory factors was increased in CORT-treated PC12 cells and serum samples of MDD patients, indicating that the inflammatory response was activated in these cells/patients and probably led to neuronal apoptosis and the occurrence of MDD.

The limitations of our study are as follows. NF-κB regulates the transcription of multiple factors, but we only investigated the effect of ketamine on MDD in relation to NF-κB expression. We did not analyze downstream molecules of the NF-κB pathway, such as caspase-3 and caspase-9. This currently limits the application of our treatment approach to preclinical studies. Further studies involving cell and animal models of MDD should investigate changes in downstream molecules of signaling pathways associated with ketamine function. Another limitation of this study is the small number of patients, and large-scale clinical studies are needed to confirm our results.
